# Kinome profiling of Arabidopsis using arrays of kinase consensus substrates

**DOI:** 10.1186/1746-4811-3-3

**Published:** 2007-02-12

**Authors:** Tita Ritsema, Jos Joore, Wilbert van Workum, Corné MJ Pieterse

**Affiliations:** 1 Utrecht University, Institute of Environmental Biology, Plant-Microbe Interactions, Sorbonnelaan 16, 3584 CA Utrecht, The Netherlands; 2 Pepscan Systems, Edelhertweg 15, 8219 PH Lelystad, The Netherlands; 3 ServiceXS, Wassenaarseweg 72, 2333 AL Leiden, The Netherlands

## Abstract

**Background:**

Kinome profiling aims at the parallel analysis of kinase activities in a cell. Novel developed arrays containing consensus substrates for kinases are used to assess those kinase activities. The arrays described in this paper were already used to determine kinase activities in mammalian systems, but since substrates from many organisms are present we decided to test these arrays for the determination of kinase activities in the model plant species *Arabidopsis thaliana*.

**Results:**

Kinome profiling using Arabidopsis cell extracts resulted in the labelling of many consensus peptides by kinases from the plant, indicating the usefulness of this kinome profiling tool for plants. Method development showed that fresh and frozen plant material could be used to make cell lysates containing active kinases. Dilution of the plant extract increased the signal to noise ratio and non-radioactive ATP enhances full development of spot intensities.

Upon infection of Arabidopsis with an avirulent strain of the bacterial pathogen *Pseudomonas syringae *pv. *tomato*, we could detect differential kinase activities by measuring phosphorylation of consensus peptides.

**Conclusion:**

We show that kinome profiling on arrays with consensus substrates can be used to monitor kinase activities in plants. In a case study we show that upon infection with avirulent *P. syringae *differential kinase activities can be found. The PepChip can for example be used to purify (unknown) kinases that play a role in *P. syringae *infection.

This paper shows that kinome profiling using arrays of consensus peptides is a valuable new tool to study signal-transduction in plants. It complements the available methods for genomics and proteomics research.

## Background

The transduction of a certain signal in the cell involves mainly the activities of kinases [1,2]. Cascades of kinases phosphorylating other kinases are set into action and lead eventually to the phosphorylation of transcription factors that are responsible for altered gene expression upon a certain stimulus. Also other processes are often regulated by phosphorylation, such as metabolic proteins and protein breakdown via the proteosome. It is estimated that one third of cellular proteins can be phosphorylated, and that by phosphorylation their activity is altered [3].

Kinome profiling aims at the assessment of the activity of kinases in a cell. It enables the parallel analysis of kinase activities and by comparison of treatments with and without a certain stimulus one can detect kinases that become activated and deactivated upon the stimulus. Kinome profiling measures kinase activities directly and is therefore a strong tool to visualize signal-transduction cascades.

The identification of proteins in extracts using proteomic tools is not directed towards the activities of the proteins. This is especially cumbersome if one studies kinases, since they are generally already present and can quickly alter their activity upon phosphorylation. On the other hand, the application of gene arrays is also not straightforward if one wants to understand signal-transduction pathways. Gene expression is only a secondary or tertiary response that occurs as a reaction to the initial signalling that has taken place. If one wants to access signal-transduction pathways, a powerful approach to do this is to measure the kinome (i.e. the complement of kinase activities).

Another advantage of kinome profiling is that it circumvents the unnatural situation created when over-expresser or inhibitor lines are used or when tagged constructs are introduced to study the involvement of a certain kinase in a process. Furthermore, kinome profiling enables the discovery of novel kinase activities in the process that is studied.

Kinome profiling on chips is a new technique that was successfully used before for mammalian cells in the discovery and characterisation of novel mechanisms in signal-transduction [4-7]. The tool used is an array of kinase substrate peptides spotted on a chip. The chip contains peptides known to be phosphorylated *in vitro *by cellular kinases and which were selected from a database of phosphorylated peptides present in Protein Kinase Research [8]. This website contains phosphorylated peptides from different organisms from bacterial, animal, plant, and fungal kingdoms that can be phosphorylated by a known or unknown kinase. For the kinome array, peptides are spotted on a glass slide and after incubation of a kinase-containing extract and radio-labelled ATP on the slide, activity of kinases can be revealed by quantifying the intensity of the radio-labelled spots. This method enables an integrated and comprehensive assessment of activities of kinases in the applied extract.

Comparing plant genomes to mammalian genomes reveals that similar families of signal transduction mediators exist in plant and mammalian systems [9]. For almost all kinase families found in mammals corresponding genes can be found in the Arabidopsis genome. For example via The Arabidopsis Information Resource (TAIR; [10]) gene families for AGC-kinases, MAP-(kinase)_1–4_, calcineurin B-interacting protein kinases, AMPK/SNF1-related protein kinases, and cell-division kinases can be found. Furthermore, various plant cascades highly homologous to mammalian signal transduction cascades have now been described [11-13].

We decided to evaluate the usefulness of kinome chips to study signalling in *Arabidopsis thaliana*. Therefore, we developed and optimized a method to assay crude cell extracts of Arabidopsis on commercially available peptide chips and we used an avirulent strain of the bacterial pathogen *Pseudomonas syringae *pv. *tomato *to show the usefulness of this approach to study signalling in plants.

## Results

### A novel peptide array can be used to visualize kinase activity of plants

**Figure 1:**
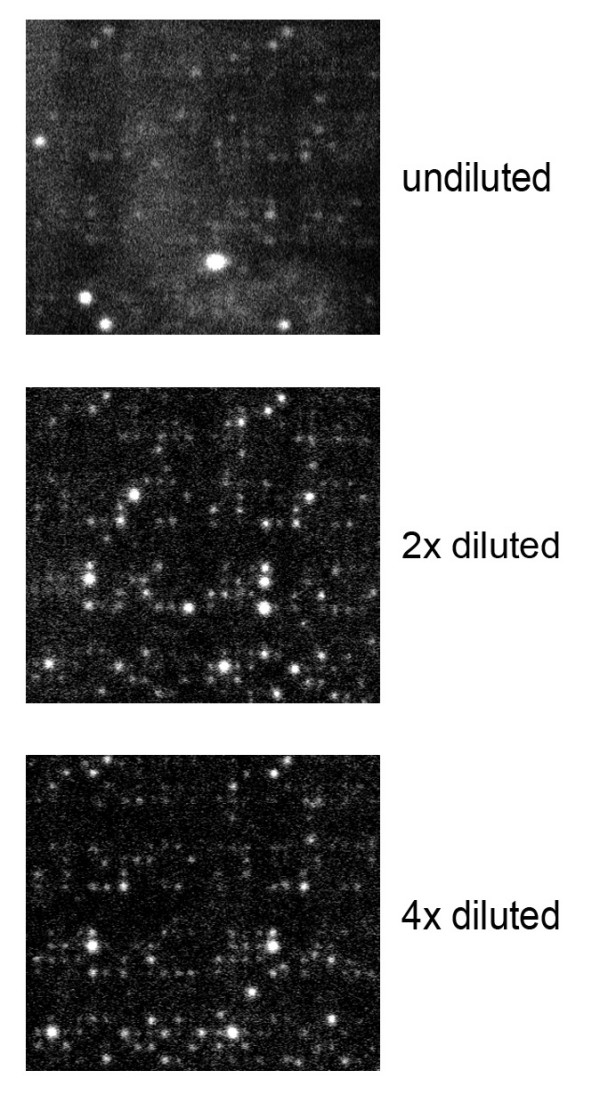
**PepChip profiles of undiluted, 2× diluted, and 4× diluted cell lysates of fresh 2- week-old Arabidopsis plants**. Chips were exposed on a phosphorimager plate for 7 days, inverse spots intensities are shown. The two technical replicates present per chip can be seen as identical patterns at the left and right half of the chip.

PepChip Kinase Trial slides containing 192 potential substrates for kinases of 8 or 9 amino acids in length were incubated with plant lysate and ^33^P-labelled ATP to visualize phosphorylation events. Cell lysates from fresh, two-week-old *in vitro *grown Arabidopsis plants were applied to a PepChip slide and kinome profiling was performed (see methods). After an exposure time of 7 days, radio-active spots were detectable on the PepChips, indicating that the corresponding peptides on the PepChip were phosphorylated by kinases present in the cellular extracts (Fig. 1).

**Figure 4:**
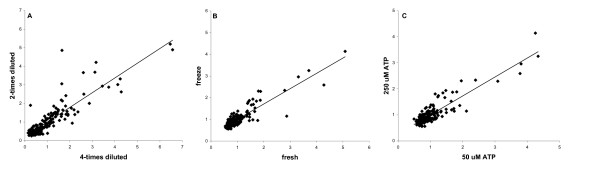
**Comparison of intensities of the same spots after different treatments**. Spots are normalized by division through the average. A trendline is drawn showing linear regression. **A**. Two- and four-times diluted lysates are compared. **B**. Fresh and frozen plants are compared. **C**. Addition of 50 μM and 250 μM non-radio-active ATP is compared.

The grey background noise in the first kinome array experiments led to a number of additional assays in which conditions were varied to optimize the kinome profiling conditions for plant extracts. One of the first parameters altered was dilution of the plant extract in fresh lysis buffer. Fig. 1 shows the 2- and 4-fold dilution of the crude cell extract in lysis buffer. Dilution resulted in a significant increase in the signal-noise ratio. Whereas spot intensities were hardly affected (Figs. 1 and 4A).

Preparing cell lysates using more lysis buffer or using two times concentrated lysis buffer (to counteract the dilution with plant liquids) did not result in a reduced background (data not shown). A possible explanation for this is that in a concentrated lysate products are present that precipitate on the slide, which can be kept in solution by dilution of the lysate. Based on these results we decided to use 4-times diluted lysate as a standard in future experiments.

**Figure 2:**
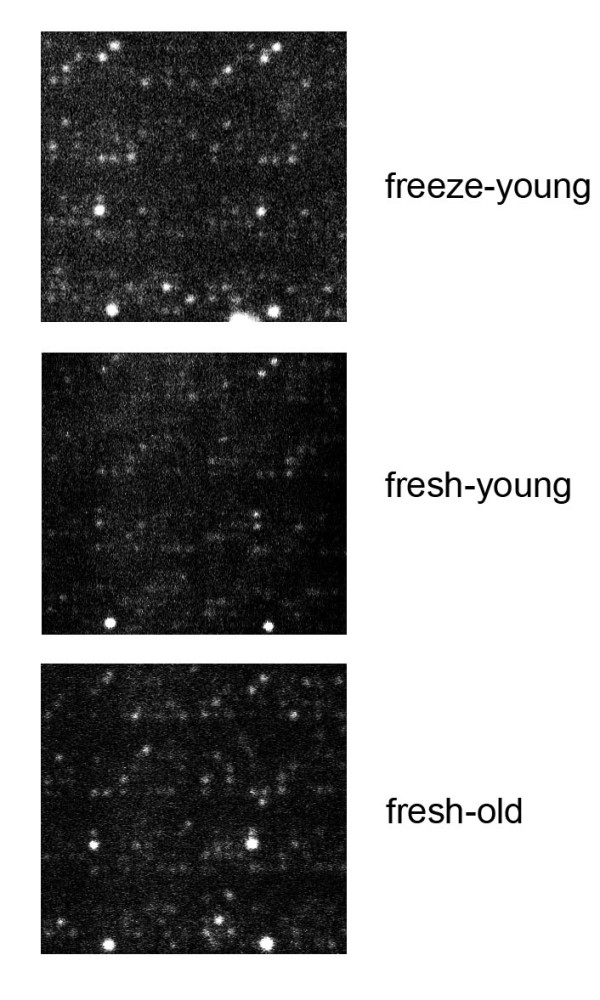
**PepChip profiles of fresh, frozen, and older plants**. Lysates of seedlings of 2-week-old (young) were made from fresh material or after snap-freezing in N_2_(l) and storage at -80°C, also lysates from fresh 5-week-old plants (old) were tested. All lysates were 4× diluted before application to the chip.

For the initial kinome profiling assays we used fresh plant material to make extracts. The use of frozen plant material for kinome profiling has the advantage that treated plant material can be stored and checked for other parameters such as protein content or gene expression before kinome profiling is performed. Therefore we tried two different freezing methods: 1) freezing fresh material and 2) freezing the cell lysate. Both were snap-frozen in N_2_(l) and stored at -80°C. Freezing cell lysates resulted in PepChips with a low signal, possibly due to reduced kinase activity (data not shown). In contrast, profiles obtained from frozen plant material resembled that of fresh leaves (Figs. 2 and 4B), showing that both fresh and frozen plants can be used to perform kinome analysis.

In addition to this we used older plants, grown for 5 weeks at short days, to make cell lysates for PepChip analysis. Also these plants resulted in comparable phosphorylation profiles on the chips (Fig. 2).

### Addition of non-radioactive ATP enhances signals

**Figure 3:**
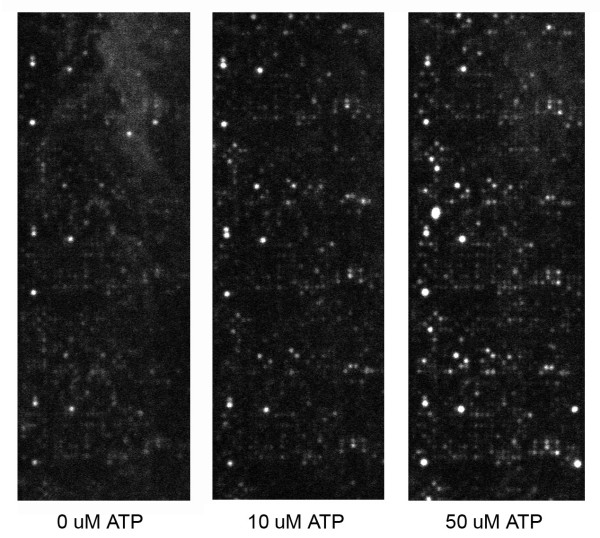
**PepChip profiles of lysates without addition of non-radioactive ATP (0 μM ATP) or with 10 μM or 50 μM ATP added**. 2-Week-old frozen plants were used and 4× diluted. The three technical replicates on these large kinome PepChips are present above each other.

The standard protocol for PepChip kinome profiling recommends adding non-radioactive ATP to an end concentration of 50 μM. Theoretically, signals could be enhanced by lowering the ratio of radioactive to non-radioactive ATP, but on the other hand detection of kinase activity can be boosted by the addition of higher concentrations of ATP. To investigate the effect of non-radioactive ATP on plant kinome profiles, 0, 10, 50, or 250 μM ATP was applied to the cell lysate. Addition of 50 μM ATP to the cell lysates strongly increased the signals on the PepChip in comparison to 0 and 10 μM of ATP (Fig. 3). Addition of 250 μM ATP yielded similar signal intensities as 50 μM ATP (Fig. 4C). Thus, addition of non-labelled ATP to the plant cell lysate enhances the PepChip signals. Apparently, the lysates do not contain enough ATP to supply the ATP needed for comprehensive measurements of kinase activity.

### Analysis of spot intensities

Spot intensities were determined using the program ScanAlyze (from the Eisen-lab) [14,15]. Grits were designed for 16 arrays of 6 spots wide and 4 spots high with a spot diameter of 10 pixels. Arrays 1 to 8 contain one replicate and 9 to 16 the second replicate. This results in spots 1–192 representing one replicate and 193–384 representing the second replicate (see also supplemental data). Spots that were overshadowed by neighbouring spots of high intensity or by background noise were flagged and their intensity was not taken into account for subsequent calculations. Spot intensities were normalized by division through the average spot intensity and for further analyses the two technical replicas present on one chip were averaged. Figure 4 shows three comparisons of spot intensities, 2- versus 4-times diluted, fresh versus frozen plants, and 50 μM ATP versus 250 μM ATP. Spot intensities are similar in all these conditions.

Up to now, analysis of PepChips in mammalian systems was done using the data as obtained from the analysis described above [4-7]. However, analysis of for example gene expression is generally performed on logarithmic values. Therefore, we decided to compare analyses of original and logarithmic values throughout the rest of the paper.

### Biological replicas and technical replicas correlate equally well

**Figure 5:**
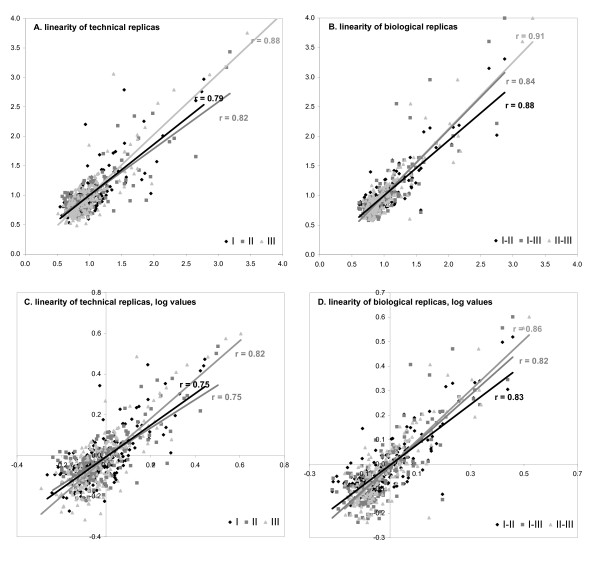
**Normalized spot intensities of three independent experiments are compared**. **A**. Technical replicas per experiment are plotted. **B**. The three experiments are pair wise compared to each other. **C**. Technical replica's of logarithmic values. **D**. Biological replica's of logarithmic values.

To measure the correlation coefficient of replicas all spot intensities were taken into account, also the outliers that were flagged during analysis (see above). The correlation coefficient (r) of the two technical replicas that are present in one slide generally ranges from 0.7 to 0.9 (linear regression, original values). As an example we show of three separate experiments these correlations (Fig. 5A, B). When calculated on original values the r for the technical replicas was 0.79 (exp I), 0.82 (exp II), and 0.88 (exp III), resulting in an explained variance (R^2^) of 63%, 68%, and 78%, respectively (Fig 5A). When calculated on logarithmic values correlation coefficient was 0.75 for exp I, 0.75 for exp II, and 0.82 for exp III (Fig 5B).

These three experiments consisted of independently grown plants that were harvested after two weeks of growth, frozen at -80°C, and separately tested on a PepChip with a 4-fold dilution of the lysate. To compare these biological replicas to each other, spot intensities were averaged over the two technical replicas and the average was compared between the three experiments (Fig. 5C, D). The correlation coefficient of these for original values is 0.88 for exp I against exp II, 0.84 for exp I against exp III, and 0.91 for exp II against exp III (Fig. 5C). For logarithmic values, these are 0.83, 0.82, and 0.86, respectively (Fig 5D).

It is striking that the correlation of technical and biological replicas is in the same range. This indicates that biological replicas are indeed representing plants in the same physiological state with similar kinase activities profiles. Surprisingly, the correlation coefficient of biological replicas tends to be even higher than that of technical replicas, possibly because the average spot intensity of the technical replicas is taken for the comparison of biological replicas, diminishing the effect of outliers.

### Kinome profiling of Arabidopsis infected with avirulent *Pseudomonas syringae *pv. *tomato*

To provide proof of concept for the usefulness of the PepChip kinase arrays for kinome profiling in plants, we decided to investigate the interaction of Arabidopsis with the bacterial pathogen *Pseudomonas syringae *pv. *tomato *DC3000, carrying the avirulence gene *avrRpt2 *[16].

**Figure 6:**
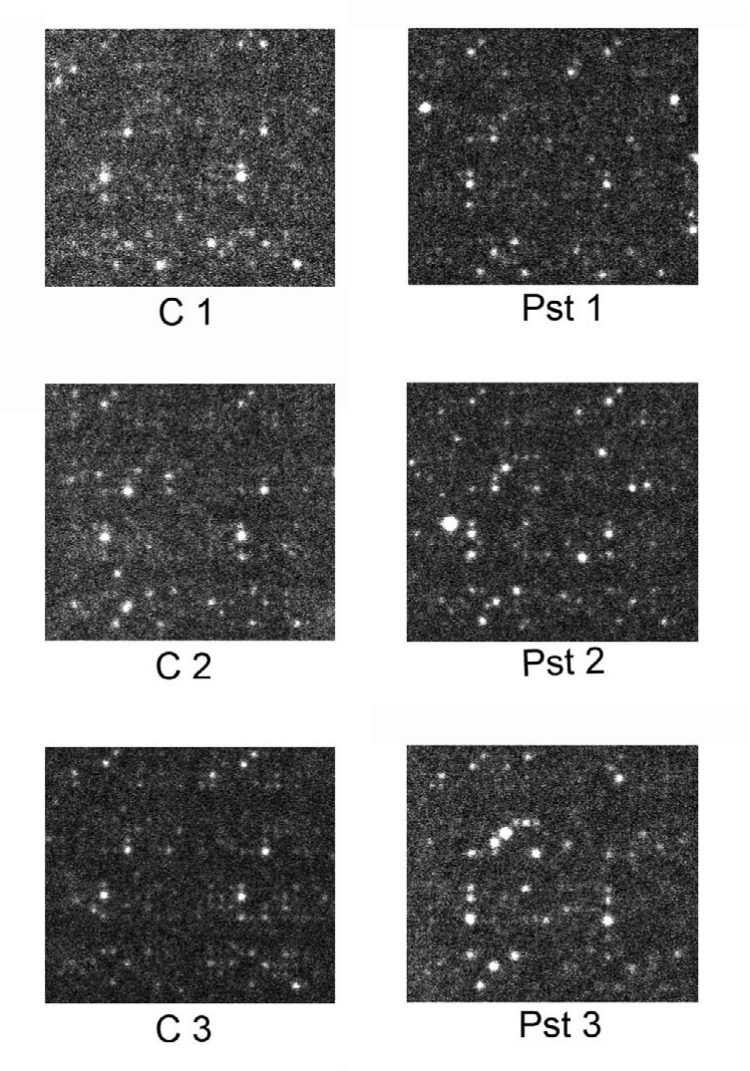
PepChip profiles of mock- (C) and *P. syringae*-treated (Pst) Arabidopsis plants for three biological replicates.

For our experiment Arabidopsis Col-0 seedlings grown on agar plates for 2 weeks were used. *P. syringae *infected and mock-infected seedlings were harvested after 1 hour for kinome profiling. Lysates of three independent experiments were tested on the PepChip to study pathogen-induced changes in the kinome (Fig. 6).

Correlation coefficients for values for the technical replicates on one chip are for the control treatments 0.81, 0.84, and 0.88; and for *P. syringae *treated plants 0.80, 0.75, and 0.68 (see supplemental data). Correlation coefficients of biological replicates, original values are for the control treatments 0.88 (exp1 – exp2), 0.84 (exp1 – exp3), and 0.91 (exp2 – exp3); for the *P. syringae *treated plants 0.91 (exp1 – exp2), 0.72 (exp1 – exp3), and 0.84 (exp2 – exp3). For logarithmic values the numbers for biological replicas are 0.83, 0.81, 0.87, 0.88, 0.75, and 0.83, respectively.

To detect differences in phosphorylation spot intensities were pair-wise compared with a Students' t-Test for the three experiments. Of the 192 peptides on the PepChip, 8 are spotting controls, so 184 peptides can be used to detect phosphorylation differences. 15 of these showed a significant differential phosphorylation pattern when original values were used with a significance cut-off of 0.05 (i.e. p < 0.05) (Table 1) [see additional file 1]. Two of these were not significantly different when logarithmic values were used and no extra spots were identified (Table 1) [see additional file 1]. Of 3 spots the phosphorylation intensity increased after *P. syringae *infection and of the resulting 12 spots the intensity decreased.

**Table 1:** Significantly differentially phosphorylated consensus peptides.

Nr.	t-Test lin.	t-Test log	P	Sequence	Kinase	ID substrate protein
12	0.0096	0.0146	▼	PSPKYPGPQ		SWISS;P05059;CMGA_BOVIN
27	0.0104	0.0168	▼	KYLASASTM	CaM kinase II	SWISS;P02687;MBP_BOVIN
50	0.0440	0.0469	▼	LSELSRRRI	ds-RNA-dep. Kinase	SWISS;P12962;IF43_YEAST
73	0.0271	0.0215	▼	PASPSPQRQ	Cyclin dep. Kinase 5	SWISS;P17599;SYN1_BOVIN
79	0.0384	0.0454	▼	LEKKYVRRD	MCSF Recept. (c-fms)	SWISS;P09581;KFMS_MOUSE
102	0.0453	n.s.	▼	KISITSRKA	ERA GTPase	SWISS;P06616;ERA_ECOLI
136	0.0023	0.0016	▼	PRRDSTEGF	Protein Kinase A	SWISS;P09201;F16P_YEAST
150	0.0441	0.0472	▲	KKAESPVKE		SWISS;P12839;NFM_RAT
152	0.0037	0.0176	▼	KTETSQVAP	Rhodopsin Kinase	SWISS;P02699;OPSD_BOVIN
153	0.0211	0.0254	▼	KRKVSSAEG		SWISS;P02316;HG14_BOVIN
164	0.0228	0.0212	▼	PVSPSLVQG	Gprot Receptor Kinase	SWISS;P08172;ACM2_HUMAN
171	0.0058	0.0065	▲	LDDQYTSSS		SWISS;P24604;TEC_MOUSE
175	0.0253	0.0230	▲	LGGGTFDIS	DnaK (Hsp70)	SWISS;P04475;DNAK_ECOLI
178	0.0005	0.0081	▼	KGATSDEED	Casein Kinase 2	SWISS;P06786;TOP2_YEAST
186	0.0418	n.s.	▼	LRRPSDQAV	Protein Kinase A	SWISS;P01126;REL_AVIRE

Lysates from 3 independent biological replicates of plants infected with avirulent *P. syringae *and mock-treated plants are put on PepChips and spot intensities were quantified (see also supplemental data). P-values derived from paired t-Tests performed on original and logarithmic normalized intensities are given; n.s., not significant (p > 0.05). Substrate proteins from which the peptides are derived and -if known- kinases phosphorylating those substrates are indicated. ▲ means higher phosphorylation intensities after treatment with avirulent *P. syringae*; ▼ means lower phosphorylation.

## Discussion

Kinome profiling using an array of consensus peptides is a novel method to measure kinome activity. Up to now few reports of the use of these arrays in mammals have been published, but the use of this method in plants is not reported. We decided to use the commercially available PepChip kinome chip and studied its usefulness in plant systems. Most substrates present on the chip have been verified in mammalian systems to be substrates for kinases. Research on plant kinases is less advanced, so it was difficult to estimate beforehand the usefulness of this system. To our surprise the available arrays can be used in a plant system with relatively minor modifications to the manual provided with the chips. One important alteration is dilution of the lysate in lysisbuffer to enhance signal to noise ratios.

The addition of ATP to the lysate increased spot intensities considerably. This indicates that ATP that is extracted from the cells is not concentrated enough to support full kinase activity. The ATP concentration in the cytosol is generally estimated to be up to 5 mM. Upon extraction this is diluted by extracellular liquids and liquids from the vacuole as well as by the lysisbuffer, and after this even an extra dilution step in lysisbuffer is performed. This probably dilutes the ATP too much to get optimal activity. Besides, the ATP concentration needed for optimal activity differs between kinases. In the assays 50 μM added ATP gave optimal activity; with no harmful effects seen when 250 μM ATP was applied, although this dilutes the radio-active ATP and, therefore, leads to less label transferred per spot.

Significant differences in phosphorylation are determined using statistics. The p-values as mentioned in Table 1 stand for the probability of observing these type of data if there was no real difference. Therefore, some of the differentially phosphorylated peptides identified are false positives (as there are false negatives that are not identified as significantly differentially phosphorylated peptides). For this reason the involvement of the kinases identified and their substrates need to be confirmed. One way to do this is to confirm the connection of the kinases or substrates identified to the process studied by expression analysis using micro-arrays. For example, in Arabidopsis 13 stress-related p70 heat-shock proteins are present that match the consensus peptide LGGGTFDIS (derived from the *E. coli *HSP70 DnaK; Table 1); these are At1g09080, At1g16030, At1g56410, At3g09440, At3g12580, At4g24280, At4g37910, At5g02490, At5g02500, At5g09590, At5g28540, At5g42020, and At5g4991. Seven of these (At1g09080, At1g16030, At3g09440, At3g12580, At5g02490, At5g09590, At5g28540) showed a highly upregulated expression after avirulent *P. syringae *infection [17]. Thus, the induction of the transcription of several heat shock proteins shows their involvement in avirulent *P. syringae *infection, which corroborates the conclusion from phosphorylation differences observed using PepChip-based kinome profiling.

Papers that report kinome profiling on PepChips used up-to-now original intensity values for data-evaluation [4-7]. This paper is the first one reporting differential phosphorylation of plant lysates on PepChips. Since phosphorylation intensity is multiplicative one could theoretically argue that it is worthwhile to evaluate logarithmic values instead of original values. For example, in the analysis of gene expression data logarithmic values are used. We compared data analysis of original and logarithmic values and show that this does not lead to major differences in correlation coefficients and identification of differentially phosphorylated peptides.

Consensus peptides can often potentially be phosphorylated by several kinases, which renders it sometimes difficult to unambiguously assign phosphorylation of a consensus peptide to activity of a certain kinase. Furthermore, substrate consensus sequences for a kinase are often not the preferred substrate of that kinase. On the PepChip many potential substrates for one kinase can be found, but not all of these will be equally well phosphorylated by that particular kinase. This was already shown for mammalian PKA, which did not phosphorylate all potential consensus substrates present on the PepChip very well, if even at all [4]. To identify which consensus substrates are phosphorylated by a certain kinase, the activity of purified kinases needs to be determined by profiling on PepChip slides.

Consensus peptides spotted on the PepChip are mainly not derived from plants. Therefore, one has to investigate which kinase phosphorylates a differentially phosphorylated peptide. One can make educated guesses as to the kinases able to phosphorylate some of the peptides. It is for example known that MBP (myelin basic protein) that is phosphorylated by mammalian MAP kinases can also be phosphorylated by plant MAP kinases [18]. Also the Kemptide, a peptide used to detect PKA activity in mammals is phosphorylated by its Arabidopsis homologue AGC2-1 [19].

A way to apply kinome profiling on the PepChip is in the purification of a kinase involved in creating an observed (differential) phosphorylation profile. For this, classical fractionation and protein purification techniques can be combined with phosphorylation profiling on the PepChip. In this way the PepChip can aid the discovery of kinases involved in the signal-transduction studied, even if these kinases were not beforehand identified as such.

Alternatively, methods such as inhibitor studies, knock-out lines and the profiling of purified kinases can be used to show a link between the treatment applied and activity of a particular kinase.

For the study of avirulent *P. syringae *infection presented here we used the small trial kinome chip, with two replicates of 192 random kinase substrates from many different organisms. For further studies a large kinome chip containing 1157 substrates is available that contains also two replicates of a general substrate set in which there are also kinase substrates present that are not necessarily linked to known proteins, such as the Kemptide, a general PKA substrate.

## Conclusion

The kinomes of plants can be studied using profiling on arrays with kinase consensus substrates. Both fresh and frozen plant material can be used, but once lysates are made freezing them diminishes kinase activities. Dilution of the lysate in lysis buffer increases the signal to noise ratio, leading to more reliable measurements of spot intensities. To get maximum phosphorylation of substrates the addition of 50 μM non-radioactive ATP is necessary.

Using Arabidopsis plants infected with avirulent *P. syringae *as a biological system we show the usefulness of kinome profiling on substrate peptide array chips. Kinome profiling is a hypothesis-generating tool and the results can for example be used for purification of specific kinases in the process studied.

This paper shows that kinome profiling using arrays of consensus peptides is a valuable new tool to study signal-transduction in plants. Kinome profiling studies kinase activities and complements the available methods of genomics and proteomics.

## Methods

### Plant growth conditions

*Arabidopsis thaliana *Col-0 plants were sown on agar plates that contained 1/2 strength MS (Murashige and Skoog with amino acids) and 0.8% plant agar and grown for 2 weeks at short days with 8 h light (200 μE·m^-2^·s^-1^; 24°C) and 16 h dark (20°C).

### Cell lysis

Cell lysate was made essentially according to the manual provided with the PepChip slides. Plants were used fresh or snap frozen in N_2_(l) and stored at -80°C. Lysates were made from 60–100 mg (fresh weight) plants that were crushed using a small pistil that fits in an Eppendorf tube in 200 μl lysisbuffer. The lysisbuffer consists of 20 mM Tris-HCl pH 7.5, 150 mM NaCl, 1 mM EDTA, 1 mM EGTA, 1% Triton X-100, 2.5 mM Na_4_P_2_O_7 _(sodium pyrophosphate), 1 mM β-glycerophosphate, 1 mM Na_3_VO_4 _(sodium orthovanadate), 1 mM NaF, 1 μg/ml leupeptin, 1 μg/ml aprotinin and is kept frozen in aliquots at -20°C. 1 mM PMSF is added fresh from a stock of 200 mM in isopropanol. The lysate was put on ice for 5 to 10 min and debris was spun down at 4°C for 10 min at maximum speed in an eppendorf centrifuge. The supernatants were filtered through a 0.2 μm filter to remove particles.

### Incubation of the lysates on chips

Activation of the lysates and incubation on the slides was performed as described in the manual provided with the PepChips. Cell lysates are activated by the addition of 12.25 μl activation mix to 50 μl of lysate. Activation mix is made fresh by adding 10 ml 50% glycerol, 0.15 ml 100 mM ATP, 0.6 ml 1 M MgCl_2_, 0.1 ml 3% Brij-35, 0.3 ml 5 mg/ml BSA.

PepChip Kinase slides (PepScan Systems, the Netherlands) were incubated with the lysate plus activation mix and 3 μl radioactive ATP (3 μCi γ-^33^P ATP, SA 1000–3000 Ci/mmol, GE Healthcare/Amersham) was added. After incubation for 2 h at 30°C in 100% relative humidity, the slides were washed twice in PBS with 0.05% Tween20, twice in 0.5 M NaCl (filtered trough a 0.2 μm filter) and twice in milliQ, after which they were placed in a phosphorimager cassette with an imager screen. After 7 days of exposure, radioactivity was quantified using a BioRad Phosphorimager with QuantityOne software.

### *P. syringae *infection

The avirulent bacterial pathogen *Pseudomonas syringae *p.v. *tomato *DC3000 (*avrRpt2*), harbouring the plasmid pV288 that contains the avirulence gene *avrRpt2 *[20], was grown overnight at 28°C in liquid LB. After centrifugation, the bacterial suspension was resuspended to an OD of 0.01 in demi water, representing 10^7 ^bacteria per ml. Bacteria were used to vacuum infiltrate seedlings that were grown on agar plates for 2 weeks. Vacuum infiltration was performed in a Speedvac for 1 min, without heating. Seedlings were washed in demi and left in demi for 1 hour prior to freezing in N_2_(l). As a control seedlings were treated equally with only demi water.

## Competing interests

TR and CP declare that they have no competing interests; JJ is manager at PepScan Systems the company that manufactures the PepChips; and WW is manager at ServiceXS, the company that distributes the PepChips.

## Authors' contributions

TR did the experiments and analyses and wrote the manuscript; JJ and WW facilitated, and guided the use of PepChip slides; and CP proposed the *P. syringae *experiments, supervised the work, and corrected the manuscript. All authors have seen and approved of the final manuscript.

## Supplementary Material

Additional File 1 **1. Raw data and analyses**. Excel file of spot intensities for all spots present on the small PepChip for the three avirulent *P. syringae *infection experiments, including the pair wise Students t-Test on original and logarithmic values to detect significant differences in intensities for the three experiments. Click here for file
